# Effects of Social Exclusion on Effortful Control and Mentalizing in relation to Borderline Personality Features

**DOI:** 10.1038/s41598-018-32775-7

**Published:** 2018-09-26

**Authors:** Momoko Sato, Peter Fonagy, Patrick Luyten

**Affiliations:** 1 Department of Clinical, Educational, and Health Psychology, University College London, London, UK; 2 Faculty of Psychology and Educational Sciences, KU Leuven, Belgium

## Abstract

The current study investigated the effects of social interactions on effortful control (EC) and mentalizing in individuals with borderline personality disorder (BPD) features. 123 nonclinical participants completed the emotional Stroop task to assess EC and the modified Reading the Mind in the Eyes Test (RMET) to assess mentalizing capacities before and after having social interactions. The Cyberball paradigm was used to generate socially inclusive and exclusive interactions. Results indicated the high BPD group made more errors on the Stroop task following exclusive social interactions than the low BPD group. The high BPD group, compared to the low BPD group, associated fewer emotional words with facial cues following inclusive social interactions but overanalysed facial cues (hypermentalizing) following the exclusive social interaction. Exclusive social interactions hindered the inhibitory capacities in individuals with high BPD features, but inclusive social interactions facilitated. Individuals with high BPD features responded to exclusive social interactions by hypermentalizing. Thus, it was found social rejection could activate cognitive-affective processes which led to hypermentalizing and impairments in EC which in combination could explain the disruptive effects on social interactions in people with BPD features.

## Introduction

Borderline Personality Disorder (BPD) is a serious and complex psychiatric disorder, which is characterized by an unstable pattern of emotional regulation, interpersonal relationships, self-image and identity, impulse control, self-harm, and suicidal tendencies^1,2^. BPD is associated with severe social functional impairments, comorbid psychiatric disorders, intensive use of treatment, and high health care costs to society due to the frequent use of health care service such as multiple referrals and admissions to Accident and Emergency Departments and inpatients units^3–5^. Interpersonal difficulties have been considered to be one of the core symptoms of BPD^6^ underlying the variety of other emotional and behavioural problems in this group^7–9^. Many manifestations of impulsivity and emotional volatility linked to BPD are observed within interpersonal contexts^7^, and the severity of these symptoms tend to increase when individuals with BPD perceive real or imagined social rejection or loss of significant others^10^. Interpersonal problems are found to be the most common triggers for serious suicide attempts in BPD patients^11^. However, there is less agreement about the cognitive and affective impairments that might play a part in mediating the impact of such hypersensitivity on their social relationships. Past literature has found mixed evidence with some studies showing enhanced^12–14^ and others have reported depleted social-cognitive capacities^15,16^ in BPD patients. But in these studies, social-cognition was not measured in the social contexts where less than adequate capacity would be considered critical- in situations of social challenges. The hypothesis which requires testing concerns the impact of social rejection on the social-cognitive capacities that would be needed to manage such a challenging social situation. One can assume that the capacity for inhibition of impulsive responding (effortful control) and the capacity for elaborating a social context for that experience (mentalizing) would be called upon in typical social interchange.

As social bonds are crucial for human survival, the drive to increase social ties and to avoid social exclusion is universal^17^. Therefore, a cognitive-affective process to detect potential rejection cues is evolutionally adaptive and essential cognitive capacities^18^. When people detect potential threats such as neglects from caregivers and rejection by significant others, emotional distress caused by rejection may lead to behavioural consequences (i.e., self-harm^19^). In response to features of even innocent social interactions, people with high rejection sensitivity may misinterpret and experience intense negative emotional arousal, which may further lead to maladaptive social behaviours such as aggression or withdrawal^20^. Although it is a fundamental capacity to detect potential rejection threats, the sensitivity and readiness to detect the threat and the response to the rejection cues vary depending on individuals’ personality^17^. Rejection sensitivity has been defined as a cognitive-affective dysfunctions characterized by anxious expectations of rejection and intense reactions in response to perceived rejection-relevant cues^21^.

BPD patients are considered to be particularly hypersensitive to potential threats when they encounter emotionally challenging interpersonal situations^22^. BPD patients tend to exhibit higher drive to belong with others^23^ and to avoid abandonment because of their intense fears they have about rejection^20^. However, they also more likely to be aggressive or impulsive, and dismissive in interpersonal situations^6,24^. This maladaptive emotional response (i.e., aggression) may lead to actual social rejection despite their high desire to be accepted by others. Many studies have supported the rejection-hypersensitivity and maladaptive response to rejection in BPD patients. In the past study^25^, BPD patients showed extreme evaluations of others such as dichotomous thinking (i.e., all good/ all bad) only in situations where there was an emotionally provoking theme such as relationship crisis. Another study found that BPD patients exhibited rejection and anger related attributions and interpretation bias in ambiguous social situations^26^. Thus, BPD patients’ hypersensitivity to rejection and abnormal response to negative interpersonal stimuli has been captured in previous studies, but the underlying dysfunction is still not well understood. One of the impairments that may explain their interpersonal difficulties in BPD patients is their social cognitive impairments^27^.

Impairments in social cognition such as mentalizing, the capacity to refer to the self and other’s intentions, are considered to contribute to the interpersonal difficulties of BPD patients^28^. Awareness and understanding of one’s own and others’ intentions which may explain behaviours are important to facilitate effective social interactions. To come to an understanding of the intention behind someone’s actions, we use both our understanding of the context placing ourselves into the situations of the other and external cues we extract from our observation of their actions. Hence, the negative cognitive biases may contribute to ineffective mentalizing in BPD patients. However, past findings regarding the mentalizing capacity of BPD patients are still mixed. Recent studies have suggested enhanced capacities to infer others’ mental states in BPD patients. It was have found that BPD patients were found in several studies to be more accurate on the Reading the Mind in the Eyes Test (RMET) than the healthy control group^12–14^. Other studies have suggested BPD patients have a lower capacity to recognize other’s emotional state in facial expressions^15^, and to discriminate negative facial expressions^16^ than healthy controls. Using the Movie for the Assessment of Social Cognition (MASC), a newly developed method to assess mentalizing, other studies have found that BPD features predicted “over-mentalizing” where they were less accurate and excessive in mentalizing^29^. Hence, it is still not clear whether BPD patients’ capacity to mentalize is enhanced or not.

Recent reviews suggested that BPD patients do not simply lose mentalizing capacity per se, but their mentalizing can be ineffective because the capacity itself can be best seen as most effective when balanced between two extremes of a number of polarities or continua^30^. The exceptional performance of BPD patients on the RMET task could be seen in terms of their imbalanced mentalizing favouring information gained from the outside rather than information about mental states derived from internal sources. BPD patients may be characterised by an excessive focus on external features of others because their capacity for computing internal states is limited. Cognitive and affective neuroscience studies have suggested that two overlapping factors contribute to the balance of mentalizing: stress/arousal and the use of attachment strategies. Authors^30^ proposed that the activation of the attachment system in response to stress is associated with ineffective mentalizing. Hence, mentalizing capacity consists of both persistence (trait) and temporal (state) aspects that can be influenced by emotional arousal and social interpersonal contexts (i.e., social rejection).

As social cognition, mentalizing, can be imbalanced in response to stress by external stimuli (i.e., social rejection), the elevated emotional arousal by stress needs to be regulated to maintain the effective mentalizing. Hence, self-regulating capacities are essential to enable healthy social interactions through balanced mentalizing. However, numerous clinical reports and research have suggested that BPD patients have impairments in self-regulating capacities, particularly effortful control (EC)^31^. EC is a temperament aspect of self-regulation capacities to override and inhibit automatic or habitual reactions in an effortful and controlled manner to give expression to more socially appropriate response^32–34^. EC is the intentional narrowing of attentional and behavioural capacities with the aim of regulating and guiding behaviour towards a specific goal^35^. It may developmentally link to processes underpinning executive function^36^ and has been shown to be relatively poor in individuals with both internalizing and externalizing problems^37^. EC composes three aspects including the ability to make actions where there is a strong tendency to avoid the action (activation control), to inhibit inappropriate response/behaviours (inhibitory control), and to focus and shift attention where it is needed to do so (attentional control)^38^. Past research has supported that a decrease in EC was associated with an increase in psychological problems in interpersonal contexts^31,39^. Impairments in EC, particularly attentional control, were associated with an increase in BPD features among student samples^40^, patients with personality disorders^41^, as well as BPD patients^42^. As BPD patients have difficulties with affect regulation^43^, their attention may focus on negative aspects when they are unable to downregulate their emotional arousal in negative social situations. They then may fail to disengage their attention from perceived negative social cues such as rejection-relevant cues. Their elevated affective reactions may make them focus excessively on rejection cues that serve see others’ behaviours as rejecting which in turn further intensifies their emotional arousal. Consistent with these assumptions, past studies have shown attentional biases in BPD patients^44,45^. To explain the link between EC impairments and interpersonal difficulties in BPD patients, we are suggesting that the combination of disturbed mental state decoding capacities, and a vulnerability of EC in response to interpersonal distress may explain the long-term social difficulties that individuals with BPD experience.

The current study aims to investigate the impact of interpersonal stress (rejection) on social cognition (mentalizing) and self- control (effortful control) capacities in individuals with BPD features. Given that past studies have found the interpersonal hypersensitivity in BPD patients, people with a high number of BPD features are expected to be slower and make more errors on a task requiring effortful control in response to social exclusion than those with few BPD features. In addition, social stress (exclusion) expected to create the tendency to overelaborate mental state of others and suggest a wider variation of expression being linked to a facial-presentation in relative to the low-stress condition (inclusion) among those with high BPD features. Hence, individuals with high BPD features, compared to people with low BPD features, are expected to hypermentalize facial stimuli (RMET) and respond slower and less accurately on the Stroop task after social ostracism relative to inclusive social interactions. To capture the effect of social distress on cognitive capacities, repeated measurements on the cognitive tasks were conducted.

## Materials and Methods

### Participants and procedure

188 nonclinical participants (132 females and 56 males; age range 18–52 years; M_age_ 23.13, *SD* 6.23) were recruited from the University College London (UCL) psychology subject pool (SONA) system to participate the two-part study. Although 188 participants completed the first part of the study (online study), some participants withdrew from the study and did not complete the second part (lab study). In total, 125 participants were invited to come to the lab to complete the second part of the study. Although the current study recruited only non-clinical participants, two participants with a diagnosis of mental illness came to the lab. Hence, their data was excluded from the analyses. Overall, participants were White/Caucasian (37.6%), African/Caribbean (5.0%), Asians (49.6%), mixed (7.1%), and others (0.7%). The study was approved by the UCL Research Ethics Committee and was performed in accordance with the UCL ethic guidelines and regulations. All participants completed voluntary informed consent forms. After participants signed up on the SONA system, the researcher sent an online survey link via e-mail. All questionnaires were randomized in order and administered using an online survey system, Qualtrics. Participating students were compensated with course credits and non-student participants were compensated with £10 after completing the study.

### Procedure

**Figure 1:**
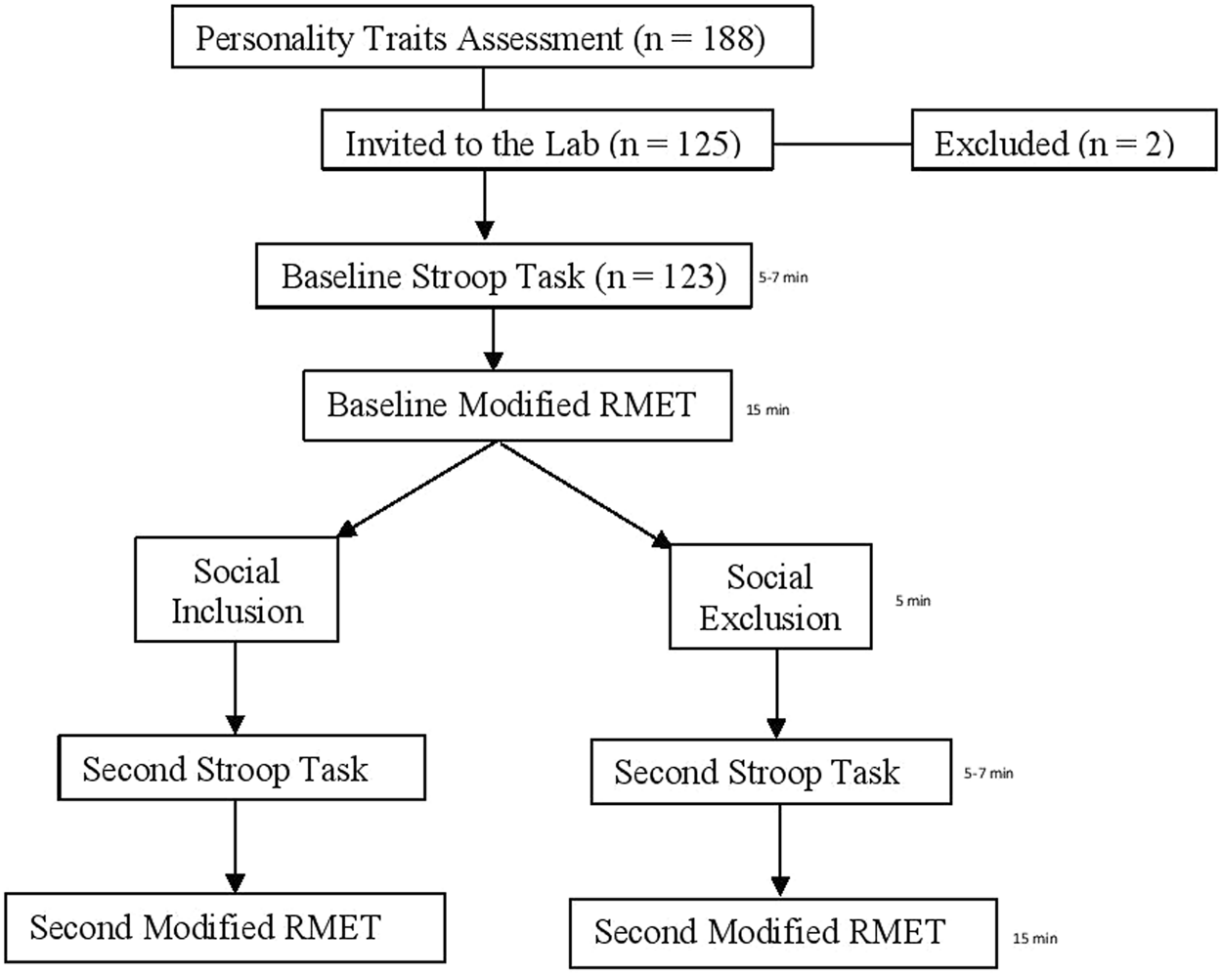
A flowchart of the sampling and study procedure.

Participants were contacted via e-mail by researchers regarding the two-part study where the first part of the study was an online study and the second part was conducted at the lab. Once they indicated interests in participating in the first part of the study, researchers sent an online link to the survey containing demographic information and personality assessments using Qualtrics. Course credits were provided once the online survey was completed. Participants were then invited to come to the lab to complete the second part of the study including four computer tasks described in detail below. When participants showed interests in participating in the second part of the study, they were asked to come to the lab within 2 weeks after the first part of the study. Arriving at the lab, participants completed the consent form describing the use of a photo in the study. Researchers then took a photo of the participants which was deleted after the study. The first computer task was the emotional Stroop task presented by Qualtrics followed by the instruction. Then participants completed the modified RMET. After the baseline assessments (Stroop and RMET), participants were randomly allocated to an inclusion/exclusion condition using the Cyberball paradigm. Following the interactions, participants were then asked to complete the last computer tasks which included the emotional Stroop task and the modified RMET (Fig. 1). After the study finished, all participants were debriefed and explained regarding the deception in the study.

### Materials

#### Personality assessment inventory-borderline features scale

Participants’ level of BPD features was assessed using the Personality Assessment Inventory-Borderline Features Scale (PAI-BOR)^46^, which is a 24-item self-report questionnaire assessing four core factors of the construct of BPD containing six items per subscale: affective instability, identity problems, interpersonal problems, and self-harm^46^. Participants were instructed to indicate the degrees of agreement to each statement using a four-point scale (0 = *false*, 1 = *slightly true*, 2 = *mainly true*, and 3 = *very true*). Past research has shown high reliability (Cronbach’s *α* = 0.93), and convergent validity with the Personality Diagnostic Questionnaire Fourth Edition-BPD Scale (PDQ4-BPD) (*r* = 0.86) in a nonclinical population^47^. The current study also showed a high internal consistency (Cronbach’s *α* = 0.87). The PAI-BOR scores in the current samples (*M* = 25.49, *SD* = 10.73) were generally consistent with the previous research^47^ (*M* = 26.71, *SD* = 14.70) in non-clinical subjects. 14 participants (12%) scored above the clinical cut-off (PAI-BOR > 38). As the number of subclinical participants (PAI-BOR > 38) was small, the high and low BPD group were formed using the median score on the PAI-BOR in the current study.

#### Reading the mind in the eyes test

The Reading the Mind in the Eyes Test (RMET)^48^ was used to assess participants’ ability to identify emotional states of others. There are total 36 black-and-white pictures of individuals’ eye-region of the face including 18 males and 18 females. Each photo was originally 15 cm × 6 cm, which was adjusted accordingly by Qualtrics on the computers. The 36 pictures were randomly divided into two groups, where the first group was presented before the social interaction task, and the second group was presented after the social interaction to avoid the habituation to the pictures. The original RMET assess the emotional inference aspect of the theory of mind. The current study modified the original RMET by adding more emotional descriptive words, which are divided into two groups. Group one contains four words that are the same from the original study to measure the accuracy. Group two contains 12 words including the words in the group 1 to quantify the level of mentalizing. If more words are selected to associate with each picture of eyes, the person is considered to be hypermentalizing. In the RMET, the number of words selected for each picture was added for all pictures to calculate the total number of selected descriptive words to quantify the level of mentalizing. The internal consistency among the total number of words selected was found to be high in the current samples (Cronbach’s Alpha 0.93).

#### Emotional stroop task

Individuals’ level of EC was assessed by an emotional Stroop task via Qualtrics. Each word written in a different colour was presented on a computer screen. When the words are written in colours other than black, participants have to select the choice indicating the colour of the word (i.e., green). When the words are written in black, they have to select the word indicating the content (i.e., music). Participants can answer multiple times if they make a mistake within 3.5 seconds. The computer will record the total time they spent to select answer choices. If a participant could not answer within 3.5 seconds, it is considered that the participant spent 3.5 seconds for the word. A list of words was selected from the study^49^ which are presented in 5 colours (Red, Blue, Purple, Green and Black). In total, there were 75 words presented in the study, and each trial contained one-word stimuli (in total 75 trials) in the task.

#### Cyberball paradigm

The Cyberball paradigm^50^ is an online ball-tossing game in which participants were told they were playing with two other participants. In reality, no other participants were involved. On the screen, there were three avatars, and two confederates’ pictures were presented. There were in total 30 ball tosses during the game among three players including participants. In the social inclusion condition, participants received a fair amount of ball tosses, which is one-third of total ball tosses (n = 10). In the exclusion condition, participants received only two ball tosses out of 30 ball tosses during the game. When participants received the ball, participants had a choice to return the ball to one of two confederates. To facilitate the cover story, researchers asked them to take a picture to share with other participants in the online game. However, the pictures were not used in the game.

#### Statistical analysis

Pearson correlation coefficients were first calculated between age and all outcome variables. As age was correlated with the RMET total score, age was controlled in the main analyses. Independent-samples t-tests were conducted to assess gender effects on the Stroop task and the RMET performance. As there was a significant gender difference on the RMET total store, gender was controlled in the analysis. Three one-way ANOVA for the baseline-assessments were conducted to assess the baseline difference in the emotional Stroop reaction time and the accuracy, and the RMET accuracy based on BPD features. A one-way ANCOVA for baseline-assessments to examine the baseline difference on the RMET total number selected between the high and low BPD group was conducted controlling for age and gender. Then a number of three-way ANOVA were conducted on the emotional Stroop reaction time, the Stroop accuracy and the RMET accuracy. A three-way ANCOVA was conducted on the RMET total score.

## Results

### Descriptive statistics and bivariate correlations

The means and standard deviation of the emotional Stroop performance (reaction time and accuracy) and the RMET performance (accuracy and a total number of words selected) in each condition are presented (see Table 1). Bivariate correlational analyses found age was significantly correlated with the total number of emotional words selected in RMET (*r* = −0.18, *p* = 0.03). Hence, age was treated as a covariate in the main analysis on the RMET.

**Table 1:** Mean scores of two groups before and after the interaction.

Variable	Before interaction		After interaction	
	Low BPD M (SD)	High BPD M (SD)	Low BPD M (SD)	High BPD M (SD)
**RMET Total**	51.99 (15.35)	51.93 (16.06)	54.47 (14.26)	54.79 (13.25)
Inclusion	52.84 (16.90)	50.48 (16.15)	56.72 (14.04)	51.77 (11.00)
Exclusion	50.39 (13.36)	52.95 (14.72)	52.21 (14.36)	58.26 (14.90)
**RMET Accuracy**	12.28 (2.41)	12.10 (2.31)	13.03 (2.28)	13.41 (2.28)
Inclusion	12.41 (2.52)	12.40 (2.01)	12.93 (2.27)	13.54 (2.43)
Exclusion	11.91 (2.17)	11.67 (2.53)	13.13 (2.32)	13.25 (2.12)
**Stroop Total**	126.98 (31.49)	121.21 (31.27)	111.72 (25.30)	102.80 (22.41)
Inclusion	135.11 (31.16)	113.88 (29.72)	118.13 (26.89)	99.45 (21.95)
Exclusion	116.76 (29.23)	127.69 (31.65)	105.52 (22.38)	107.00 (22.67)
**Stroop Accuracy**	66.68 (5.80)	65.53 (4.80)	65.47 (6.35)	65.31 (5.83)
Inclusion	66.44 (5.69)	66.74 (4.20)	63.90 (6.62)	67.00 (4.78)
Exclusion	66.97 (5.99)	64.28 (5.15)	67.03 (5.76)	63.15 (6.42)

*Note*. PAIBOR = Personality Assessment Inventory-Borderline Feature scale. RMET = Reading the Mind in the Eyes Test.

### Baseline differences

An independent-samples t-test found a significant gender difference on the RMET total scores; t(183) = – 2.57, *p* = 0.01. Male participants associated less emotional words with facial stimuli (*M* = 47.47, *SD* = 16.73) than female participants (*M* = 53.85, *SD* = 14.88). There was no gender difference in the Stroop performance and the RMET accuracy. Results indicated there was no significant baseline difference between low and high BPD group in the emotional Stroop and the RMET performance.

### Main analysis

**Figure 2:**
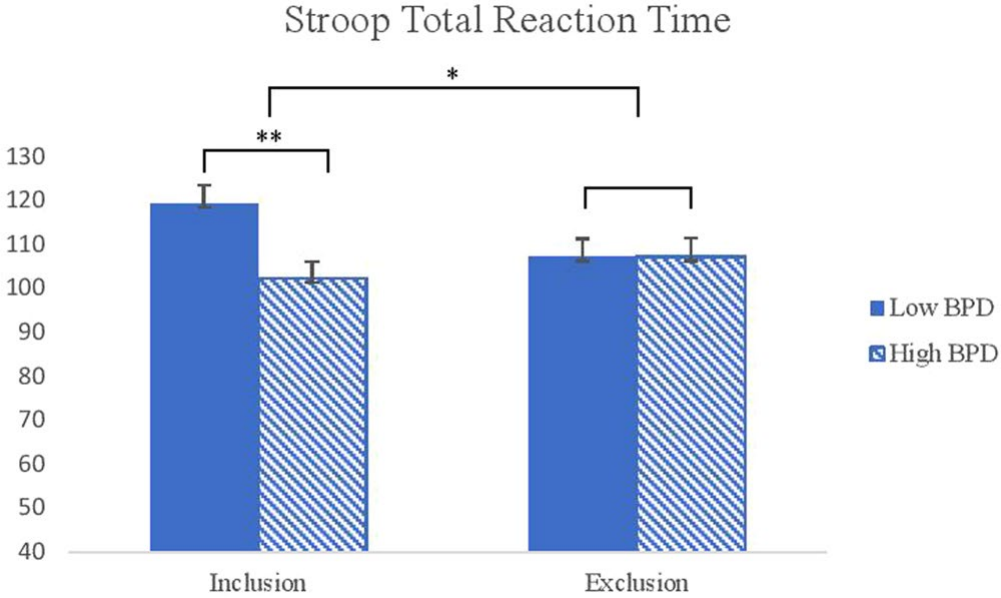
Interaction effects between conditions and the level of BPD features on the total reaction time of the emotional Stroop task. *Note*. BPD = Borderline Personality Disorder. ^*^p < *0.05*, ^**^P < *0.01*.

A three-way ANOVA on the emotional Stroop total reaction time revealed a significant BPD features by condition interaction (*F*(1,117) = 4.33, *p* = 0.04, η_p_^2^ = 0.04). Individuals with high BPD features were significantly faster than those with low BPD features after inclusive interactions. However, there was no difference between the low and high BPD groups after exclusive interactions (see Fig. 2). There was also a significant main effect of time (*F*(1, 117) = 18.66, *p* < 0.001, η_p_^2^ = 0.14) indicating that participants responded faster in the Stroop task after than before they completed the interaction task. Further, there was a significant main effect of BPD features (*F*(1,117) = 4.31, *p* = 0.04, η_p_^2^ = 0.04) where those with high BPD features was faster (*M* = 104.76, *SD* = 2.99) than individuals with low BPD features (*M* = 113.31, *SD* = 2.93).

**Figure 3:**
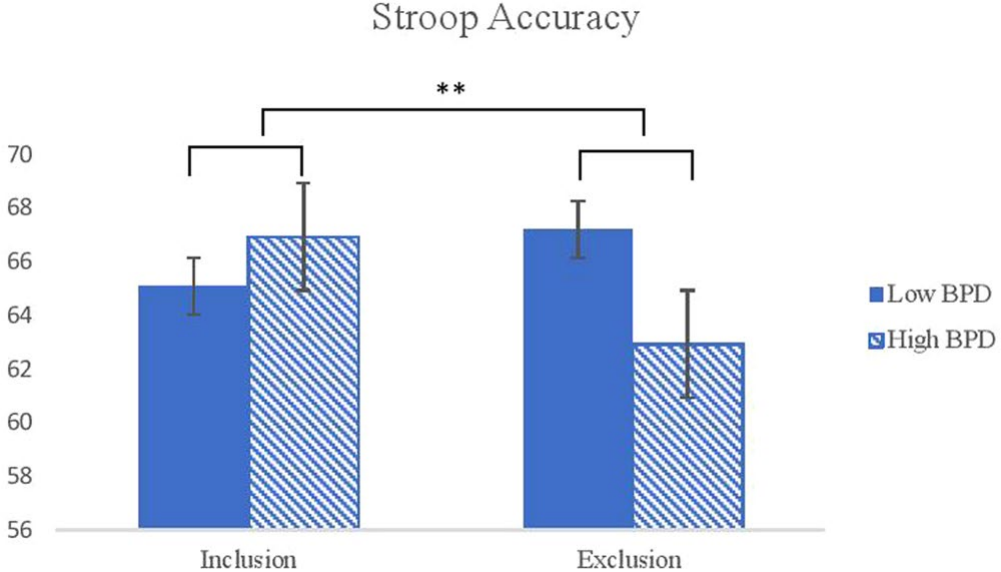
Interaction effects between conditions and the level of BPD features on the emotional Stroop task accuracy. *Note*. BPD = Borderline Personality Disorder. ^*^p < *0.05*, ^**^P < *0.01*.

A three-way ANOVA on the emotional Stroop accuracy revealed a significant condition by BPD features interaction (*F*(1, 115) = 10.16, *p* = 0.002, η_p_^2^ = 0.08) where the high BPD group was more accurate than the low BPD group after social inclusion, but less accurate than the low BPD group after social exclusion (see Fig. 3).

**Figure 4:**
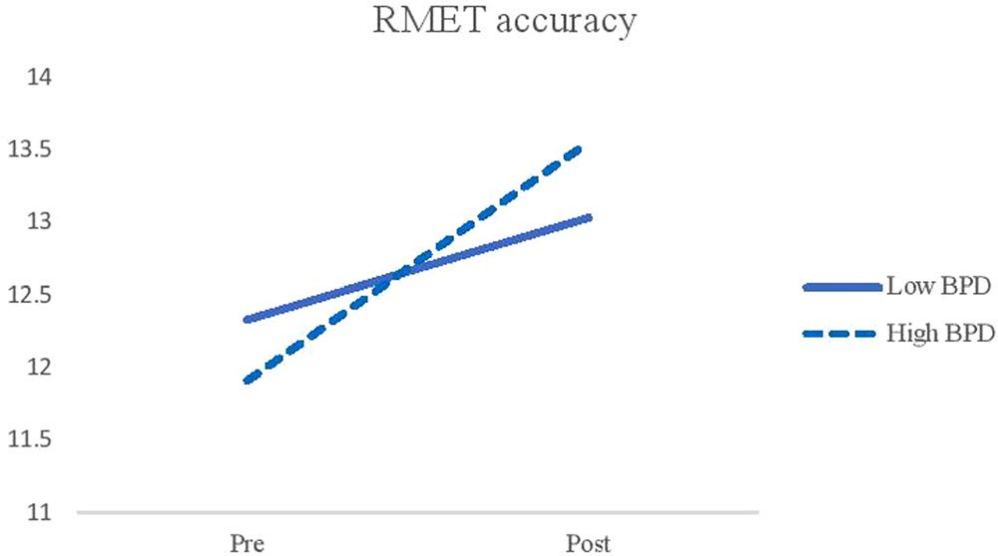
Interaction effects between time and the level of BPD features on the RMET accuracy. *Note*. BPD = Borderline Personality Disorder. RMET = Reading the Mind in the Eyes Test.

Results on the RMET accuracy found a significant main effect of time (*F*(1, 116) = 28.64, *p* < 0.001, η_p_^2^ = 0.20) and time by BPD features interaction (*F*(1, 116) = 4.59, *p* = *0.03*, η_p_^2^ = 0.04) suggesting that the low BPD group was more accurate than the high BPD group on the RMET before social interactions. However, the high BPD group became better than the low BPD group after social interactions. (see Fig. 4).

**Figure 5:**
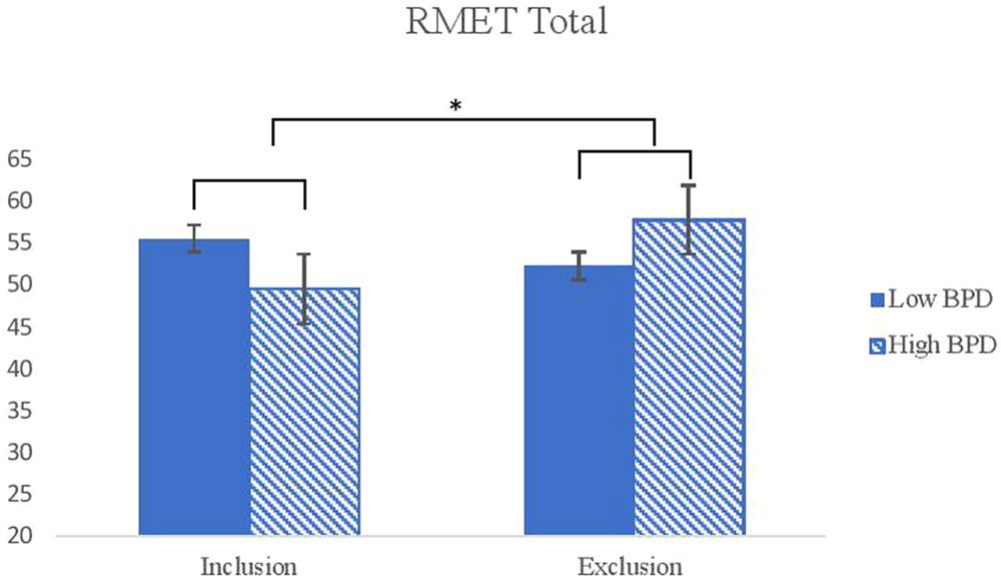
Interaction effects between conditions and the level of BPD features on the RMET total score. *Note*. BPD = Borderline Personality Disorder. RMET = Reading the Mind in the Eyes Test. ^*^p < *0.05*, ^**^P < *0.01*.

A three-way ANCOVA on the RMET total revealed a significant condition by BPD features (*F*(1, 110) = 7.21, *p* = 0.008, η^2^ = 0.06) indicating that the high BPD group associated less emotional words than the low BPD group in the inclusion condition. However, participants with high BPD features associated more emotional words with facial stimuli than participants with low BPD features in the exclusion condition (see Fig. 5).

## Discussion

The current study investigated the effect of induced interpersonal distress (social exclusion) on the cognitive capacities including the EC and mentalizing for individuals with BPD features. Results revealed that there was a difference in EC measured by the emotional Stroop task for those with high BPD features compared to those with low BPD features. Although many past studies found that BPD patients were slower than control participants with the emotional Stroop task^44,45^, current subjects with high BPD features responded faster than those with low BPD features indicating that they were more impulsive. However, there was no difference in accuracy between the low and high BPD groups suggesting that the EC in the high BPD group was not impaired compared to those with low BPD features. Although many studies had suggested that BPD patients impaired mentalizing capacities^15,16,28^, those with high BPD features did not significantly differ from those with low BPD features in the RMET accuracy and the total number of words associated with the pictures of eyes before social interactions.

After introducing virtual social interactions by the Cyberball, people became faster in the emotional Stroop task. This is most likely due to practice effects as the stimuli used in the task before and after the social interactions were same. Further, the accuracy of the emotional Stroop task decreased after the social interaction task which may be due to the fatigue effect. It was found that the different type of social interaction impacted on EC differently for those with BPD features. Individuals with low BPD features responded faster and more accurate after they were excluded than included in the interactions. On the other hand, individuals with high BPD features responded slower and less accurate following the exclusion compared to inclusive interactions. This suggests that the experience of social exclusion interfered with EC only for those with high BPD features. Negative social interactions may have activated their attachment system which increased emotional arousal. Consequently, their cognitive process such as abilities to control for interfering stimuli was disturbed, which was shown in the slower reaction time and decreased accuracy among individuals with high BPD features.

Everybody improved in mentalizing capacity after social interactions compared to their baseline in general, but the improvement of the performance on the mentalizing task after the social interaction was particularly bigger for those with high BPD features. This may suggest that social interactions activate their social cognition to imagine others’ mental states in general, but particularly for those with high BPD features. When those with high BPD features encountered positive social interactions, they associated less emotional words to assess the mental states of the person through eyes compared to those with low BPD features. However, they associated more emotional words to explain others’ mental state than those with low BPD features when they were rejected in the social situations. In other words, they over-analysed the possible mental states of others when they experienced social exclusion. This suggests that negative social interactions activate their attachment system, which elevates emotional arousal to gives the alarm to take actions to avoid or fix the negative situations. Their strategy is to overestimate all the possible mental states of others to prepare further actions, which may lead them to hypermentalize. This is consistent with the previous finding where BPD patients were excessive in mentalizing^29^.

There are a few limitations in the current study. First, words and colour used in the emotional Stroop task before and after having the social interactions were same. It enabled participants to habituate to stimuli which may contribute to decreased accuracy. Second, the current study recruited only a nonclinical population. Some of the findings regarding the emotional Stroop task contradict to past literature within the clinical population. This may be because of the difference in severity of BPD features. Given that there were only 14 participants (12%) in the current samples who scored above the clinical cut-off on the PAI-BOR scale, there was a limited variance of BPD features. To increase the generalizability to a clinical population, future study should replicate the study within the clinical population. Third, some researchers have criticized the Cyberball paradigm for its lack of ecological validity^51^. However, most participants believed that the game was played with real people; hence, they felt negative emotions in the exclusion condition. In the future study, another behavioural paradigm using confederates should be adapted to increase the ecological validity of the manipulation. Fourth, the conceptualization of hypermentalizing in the current study has not been tested for consistency with other measurements. However, it was difficult to measure as there is no standardized measurement for hypermentalizing. Although the current study has shown a high internal consistency, future research needs to be conducted to validate the current methodology. Fifth, the current study used self-report measurements in a small sample to capture BPD features. Self-report measurements have been criticized as they were vulnerable to biases (i.e., response bias) and relied on information that was consciously available to the participants (the introspective limitation)^52^. However, the current selected the PAI-BOR as it has been widely acknowledged for its clinical utility and substantial psychometric evaluations^53^. Also, it is well validated as it has demonstrated sufficient internal consistency, test-retest reliability, and convergent^54^ and concurrent^55^ validity. Last, although gender was controlled in the analyses, imbalanced gender in the current sample may limit to capture gender effects. Effects of gender are particularly important in investigating BPD features as BPD is more prevalent in female than male^1^.

The current study has supported that social distress influenced EC and mentalizing for individuals with high BPD features differently from those with low BPD features. Hence, this study provides evidence that social interactions impact on social cognition differently for those with high BPD features. Further, the current study was the first study to capture the hypermentalizing after exclusive social interactions by using the modified RMET in people with BPD features.
